# Relative Contributions of Soil and Litter Properties to Soil Microbial Community Variations During the Restoration of Larch Plantations to Mixed Forests

**DOI:** 10.3390/microorganisms13102359

**Published:** 2025-10-14

**Authors:** Zilu Wang, Yiping Lin, Kefan Wang, Xin Fang, Nuo Li, Cong Shi, Fuchen Shi

**Affiliations:** 1 College of Life Sciences, Nankai University, Weijin Road 94, Tianjin 300071, China; 2120241517@mail.nankai.edu.cn (Z.W.); 2120231449@mail.nankai.edu.cn (Y.L.); kfwang3333@163.com (K.W.); 1120210521@mail.nankai.edu.cn (X.F.); 2120231448@mail.nankai.edu.cn (N.L.); 2 School of Environmental Science and Engineering, Tiangong University, Binshui West Road 399, Tianjin 300387, China; shicong@tiangong.edu.cn

**Keywords:** forest restoration, soil depth, restoration stages, soil and litter properties, soil bacterial and fungal communities

## Abstract

The ecological restoration process of larch plantations to mixed forests contributes to enhancing the stability and functionality of forest ecosystems, with soil microbes playing a crucial role in this process. To elucidate the changes in soil microbial communities during this transition and their relationships with soil and litter properties, the study used 16S/ITS rRNA high-throughput sequencing to investigate the diversity and composition of soil bacterial and fungal communities at two soil depths across four restoration stages, and further quantified the relative contributions of soil and litter properties to variations in microbial community structure. The results indicated that bacterial and fungal α-diversity remained relatively stable in the topsoil but varied significantly across restoration stages in the subsoil (p<0.05), with the highest levels observed during the broadleaf species invasion stage. Fungal community structure demonstrated greater sensitivity to the restoration process, whereas bacterial communities showed stronger spatial dependency. Variance partitioning analysis revealed that soil properties were the main contributors to the variations of bacterial and fungal communities, accounting for 41% and 28% of the total variance, respectively. Fungal communities were more closely associated with litter properties than bacterial communities. Redundancy analysis combined with hierarchical partitioning further revealed that soil available phosphorus (AP) and total nitrogen (TN) were key factors explaining the variation in both bacterial and fungal communities. Additionally, litter total nitrogen (LTN) also emerged as an important factor affecting soil fungal communities. These findings provide critical microbiological evidence for accelerating the forest restoration in Northeast China through soil fertility management and regulation of litter inputs.

## 1. Introduction

Natural forest ecosystems are the structurally complex and biologically diverse terrestrial systems, providing vital ecological services such as climate regulation, carbon sequestration, and soil and water conservation [1]. Accounting for over one-third of China’s total forest resources, Northeast China plays a crucial role not only in the country’s carbon cycle but also in stabilizing the global climate system. However, due to the combined impacts of war, natural wildfires, and timber harvesting, forest cover and ecological functionality in this region have steadily declined [2]. *Larix gmelinii*, recognized for its cold tolerance, rapid growth, and high-quality timber, is a major species used in plantation forestry [3]. Consequently, to address biodiversity loss, restore regional ecological functions, and meet socioeconomic development needs, large-scale larch plantations were established in Northeast China [4]. Despite their initial success, these monoculture plantations have led to ecological simplification and environmental homogenization [5], contributing to reduced plant diversity and impaired ecosystem functioning. Against this ecological backdrop, achieving ecological restoration in larch plantations, transforming the species composition of planted forests, and enhancing the stability and functionality of forest ecosystems have become widely discussed topics.

Soil microorganisms significantly influence ecosystem functions by driving critical ecological processes such as carbon and nitrogen cycling, energy flow, organic matter decomposition, and mycorrhizal symbiosis [6,7]. Some exhibit high plasticity and environmental adaptability [8]. The restoration of plantations is typically accompanied by pronounced changes in the type, biomass, and makeup of aboveground vegetation. These shifts influence the diversity and structural dynamics of belowground microbial communities by altering resource inputs like litter deposition, root exudates, and soil organic matter [9]. Research has shown that the restoration process from monoculture plantations to more complex mixed forest communities increases plant diversity, enhances rhizosphere carbon input, and exerts positive effects on microbial biomass and activity [10,11]. Soil depth is another key factor influencing microbial community composition and diversity [12]. Nutrient levels, moisture conditions, and soil physical structure all vary with depth [9]. This vertical heterogeneity shapes distinct microhabitats that influence microbial community dynamics. Current understanding remains limited regarding how soil microorganisms respond to larch plantation restoration processes and soil spatial patterns. Therefore, it is imperative to explore microbial community distribution across both restoration stages and soil depths to better understand their ecological dynamics.

In forest ecosystems, variations in soil and litter properties driven by shifts in plant communities are key factors regulating soil microbial communities [13,14]. The physical structure [15], pH [16], carbon availability [17], and nutrient status of soil have all been shown to significantly influence both bacterial and fungal communities [18]. Aside from soil factors, litter plays a pivotal role in driving nutrient turnover, and its input traits exert significant regulation over belowground ecological processes. The quality and chemical composition of litter directly determine the energy and nutrient supply during decomposition. By altering the soil microenvironment and resource availability, litter further shapes the structure and function of microbial communities [14,19]. For instance, litter carbon emerges as a key factor influencing soil microbial community dynamics during subtropical forest succession [20], while in the long-term restoration of Masson pine plantations, litter phosphorus content significantly influences changes in soil bacterial communities [21]. Despite these insights, most existing studies have predominantly focused on the individual effects of soil properties on microbial communities [22]. Further investigation into the influence of litter properties on soil microbial communities, as well as their relative contributions alongside soil properties in shaping microbial community structure, will offer a more comprehensive scientific basis for developing management strategies aimed at enhancing the ecological restoration efficiency of larch plantations through the regulation of belowground microbial processes.

During the long-term process of natural regeneration, the planted larch forests in the Langxiang National Nature Reserve have interacted with native regional tree species. Over time, the dominance of larch has gradually declined as it is progressively replaced by these native species, leading to the formation of a mixed coniferous–broadleaf forest community dominated by broadleaf species such as *Juglans mandshurica*, *Ulmus davidiana*, and *Syringa reticulata*. We focus on this valuable ecological restoration chronosequence, selecting four typical stages of larch plantation restoration and identifying representative forest stands for study. We investigated the diversity and composition of soil bacterial and fungal communities at two soil depths across these four restoration stages, explored the driving factors associated with soil and litter properties, and quantified their relative contributions to microbial community variation. Three main predictions were developed: (1) the diversity and composition of both bacterial and fungal communities vary across restoration stages and soil depths; (2) soil properties play a primary role in driving the structural variations of both bacterial and fungal communities, as litter may affect most microbial communities by altering soil conditions; (3) comparatively, soil properties exert a stronger regulatory influence on bacterial communities, whereas litter properties have a more pronounced effect on fungal communities.

## 2. Materials and Methods

### 2.1. Study Area

**Figure 1:**
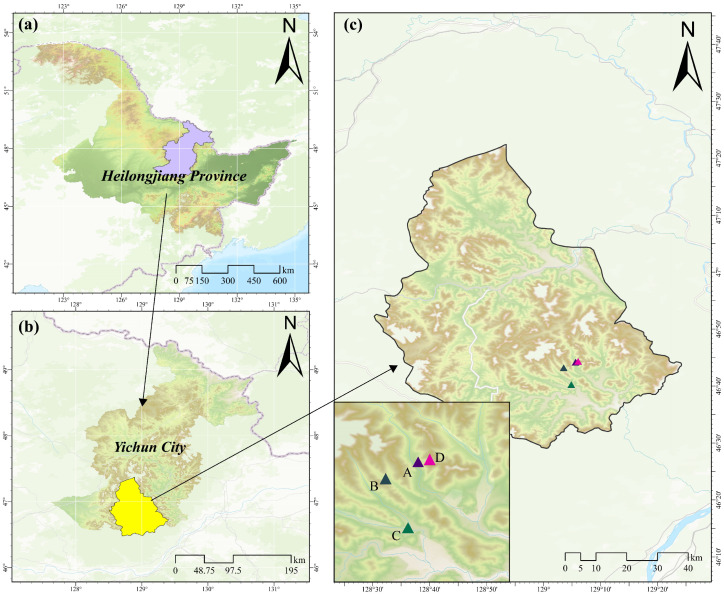
Location of the study area and sampling sites. (**a**) Heilongjiang Province, China; (**b**) Yichun City; (**c**) sampling sites. A, larch plantation stage; B, broadleaf species invasion stage; C, conifer–broadleaf competition stage; D, broadleaf dominance stage. The base map acquisition and figure creation were performed using ArcGIS Pro (v3.4.3).

The study was conducted in the Langxiang National Nature Reserve (46°31′–46°49′ N, 128°55′–129°15′ E), located at the southern foothills of the Lesser Khingan Mountains in Langxiang Town, Yichun City, Heilongjiang Province (Figure 1a,b). The climate in this region can be classified as a temperate humid continental climate, marked by four clearly defined seasons. Its mean annual temperature is 0.9 °C, and the mean annual precipitation reaches 623 mm [23]. The zonal vegetation within the reserve is temperate mixed coniferous–broadleaf forest, dominated by *Pinus koraiensis* [24].

In the 1950s, large-scale larch plantations were established within the reserve. However, these larch stands were closely interspersed with patches of natural, primary forest, allowing native tree species to persist as a seed source. Over the course of long-term natural regeneration, the planted larch forests interacted with native species. The broadleaf species exhibited strong growth and developmental vigor, leading to increased population densities and enhanced competitive strength within the forest community. Moreover, the original soil and moisture conditions of the site were more favorable to the growth of these broadleaf species, giving them a competitive advantage over larch in terms of spatial and nutrient resource allocation. This dynamic facilitated the gradual immigration of broadleaf species, and the larch plantations progressively transitioned into mixed communities dominated by key native species such as *J. mandshurica*, *U. davidiana*, and *S. reticulata*.

### 2.2. Field Investigation and Sampling

The forest restoration process was categorized into four representative stages: (A) larch plantation stage, (B) broadleaf species invasion stage, (C) conifer–broadleaf competition stage, and (D) broadleaf dominance stage (Figure 1c). Fieldwork was conducted in August 2024. Based on historical records of the reserve, representative stands for each stage were selected according to species composition, species importance value, growth situation, and understory regeneration, ensuring that each stand was in a relatively stable phase of the target restoration stage. Three 20 × 20 m replicate plots were set up per forest stand under similar site conditions (elevation, slope, aspect, etc.). A full tree census was conducted within each plot, and basic information and specific characteristics of the sample plots are shown in Table S1.

Soil and litter sampling followed the five-point sampling method. Soil samples were collected from the 0–20 cm (Topsoil, T) and 20–40 cm (Subsoil, S) layers, respectively, while litter samples were gathered from the ground surface at the corresponding five sample points. Litter samples were oven-dried, ground, and sieved (100-mesh). For each plot, five soil samples from the same layer were homogenized and split into two aliquots: one was stored at −80 °C for high-throughput sequencing, and the other was air-dried, ground, and sieved (60-mesh) for physicochemical property determination.

### 2.3. Analysis of Soil and Litter Properties

The organic carbon and total nitrogen content in the litter (LOC, LTN) and soil (SOC, TN) was determined via dry combustion using a Vario MICRO Cube elemental analyzer (Elementar, Langenselbold, Germany) [25]. The molybdenum–antimony anti-spectrophotometric method was employed to determine the total phosphorus content in both litter (LTP) and soil (TP) [14,26]. Soil ammonium nitrogen (NH_4_^+^-N) and nitrate nitrogen (NO_3_^−^-N) were analyzed by the potassium chloride extraction–indophenol blue colorimetric method and the phenol disulfonic acid spectrophotometric method, respectively [25,27]. Available phosphorus (AP) was extracted using the hydrochloric acid–ammonium fluoride method and determined by the molybdenum blue colorimetric method [28]. Soil pH was measured by the electrode method employing a water-to-soil ratio of 2.5:1 [12].

### 2.4. DNA Extraction, PCR, and High-Throughput Sequencing

Total microbial DNA was extracted from 0.5 g of fresh soil using the ALFA-SEQ Magnetic Soil DNA Kit. The purity and concentration of the DNA were assessed with a Nanodrop One spectrophotometer (Thermo Fisher Scientific, Waltham, MA, USA). The extracted DNA was used as a template to amplify the V4 region of the bacterial 16S rRNA gene with primers 515F (5′-GTGCCAGCMGCCGG-3′) and 806R (5′-GGACTACHVGGGTWTCTAAT-3′). The ITS1 region of the fungal ITS rRNA gene was amplified using primers ITS5-1737F (5′-GGAAGTAAAAGTCGTAACAAGG-3′) and ITS2-2043R (5′-GCTGCGTTCTTCATCGATGC-3′). Purified PCR products were used to prepare sequencing libraries, which were subsequently sequenced on an Illumina Nova 6000 platform (PE250) (Guangdong Magigene Biotechnology Co., Ltd., Guangzhou, China).

### 2.5. Sequencing Data Analysis

Raw sequencing data were quality-controlled using Fastp (v0.14.1), and primers were removed with Cutadapt (v4.9), resulting in high-quality clean reads. And Usearch was utilized to merge these reads into Raw Tags based on overlapping regions. After quality filtering, the resulting Clean Tags were clustered into OTUs at 97.0% similarity using Usearch (v10.0). Representative sequences of bacterial and fungal OTUs were taxonomically classified by alignment against the SILVA (v138) and UNITE (v8.0) databases, respectively.

### 2.6. Statistical Analysis

Significant differences in litter properties across restoration stages and in soil properties among restoration stages within the same soil layer were assessed using one-way ANOVA, followed by LSD post hoc tests (p<0.05). Two-way ANOVA was used to assess the effects of restoration stage, soil depth, and their interaction on soil properties, microbial α-diversity, and phylum-level relative abundances. Richness, Simpson, and Shannon indices were calculated using the “vegan” package (v2.6-10) in R (v4.4.3). Principal coordinates analysis (PCoA) based on Bray–Curtis distance was performed using the same software package to visualize variations in microbial community structure. The effects of restoration stage and soil depth on microbial community structure were tested using PERMANOVA. Linear discriminant analysis effect size (LEfSe) was conducted on the Magigene Gene platform (http://cloud.magigene.com; accessed on 26 June 2025) to identify microbial taxa that differed significantly across restoration stages (LDA > 4.0, p<0.05). Prior to further analysis, collinearity among environmental variables was examined, and variables with a variance inflation factor (VIF) ≥10 were excluded. Variance partitioning analysis (VPA) was performed using the “vegan” package (v.2.6-10) to quantify the relative contributions of grouped soil and litter properties to microbial community variation. Redundancy analysis (RDA) was applied to explore the relationships between microbial communities and both soil and litter properties. To further determine the individual explanatory power of each variable, hierarchical partitioning (HP) was conducted using the “rdaccaa.hp” package (v1.1-1), followed by significance testing [29]. To examine the relationships among α-diversity, phylum abundance, and soil/litter properties, a Spearman correlation analysis with n = 24 was employed. Results of PCoA, VPA, RDA, hierarchical partitioning, and Spearman correlation analysis were visualized using ggplot2 (v3.5.1).

## 3. Results

### 3.1. Soil and Litter Properties

**Figure 2:**
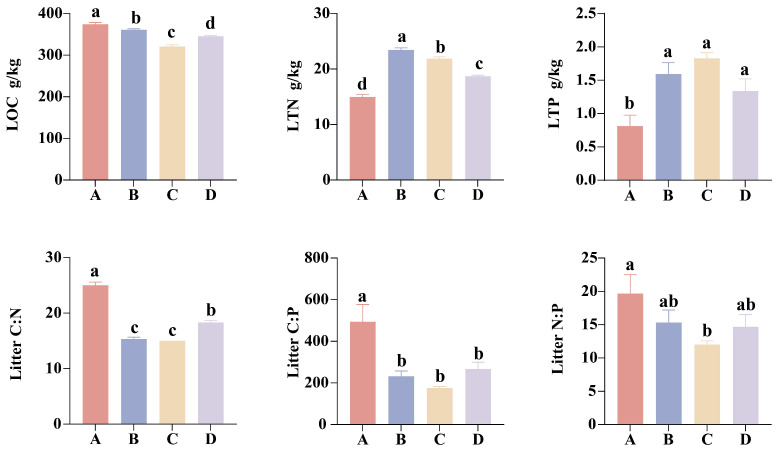
Changes in litter properties at different restoration stages. Different lowercase letters indicate significant differences in litter properties among restoration stages (p<0.05).

Significant differences in LOC, LTN, and litter C:N were observed among different restoration stages (p<0.001, Figure 2, Table S2). The larch plantation stage exhibited the highest levels of LOC (374.43 g/kg) and litter C:N (25), both of which followed a decreasing-then-increasing trend throughout the restoration process. In comparison, LTN peaked at 23.43 g/kg during the broadleaf species invasion stage (Figure 2, Table S2).

**Figure 3:**
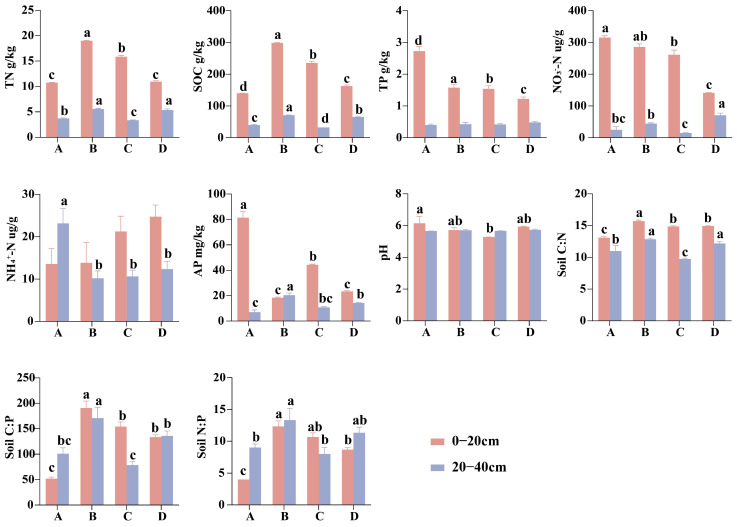
Changes in soil properties of the 0–20 and 20–40 cm soil layers at different restoration stages. Different lowercase letters indicate significant differences in soil properties among different restoration stages within the same soil layer (p<0.05).

With the exception of soil NH_4_^+^-N and pH, all other measured soil properties were significantly influenced by the restoration stage, soil depth, and their interaction effects (Table S3). Overall, the topsoil had higher soil properties than the subsoil. Across different restoration stages, both soil layers reached their highest SOC, TN, and stoichiometric ratios during the broadleaf species invasion stage, followed by a decline as restoration progressed. In the topsoil, the contents of TP (2.7 g/kg), NO_3_^−^-N (316 ug/g), and AP (81 mg/kg) were highest during the larch plantation stage and exhibited a decreasing trend over the restoration process, whereas the subsoil showed an opposite pattern (Figure 3, Table S3).

### 3.2. Diversity of Soil Bacteria and Fungi

**Figure 4:**
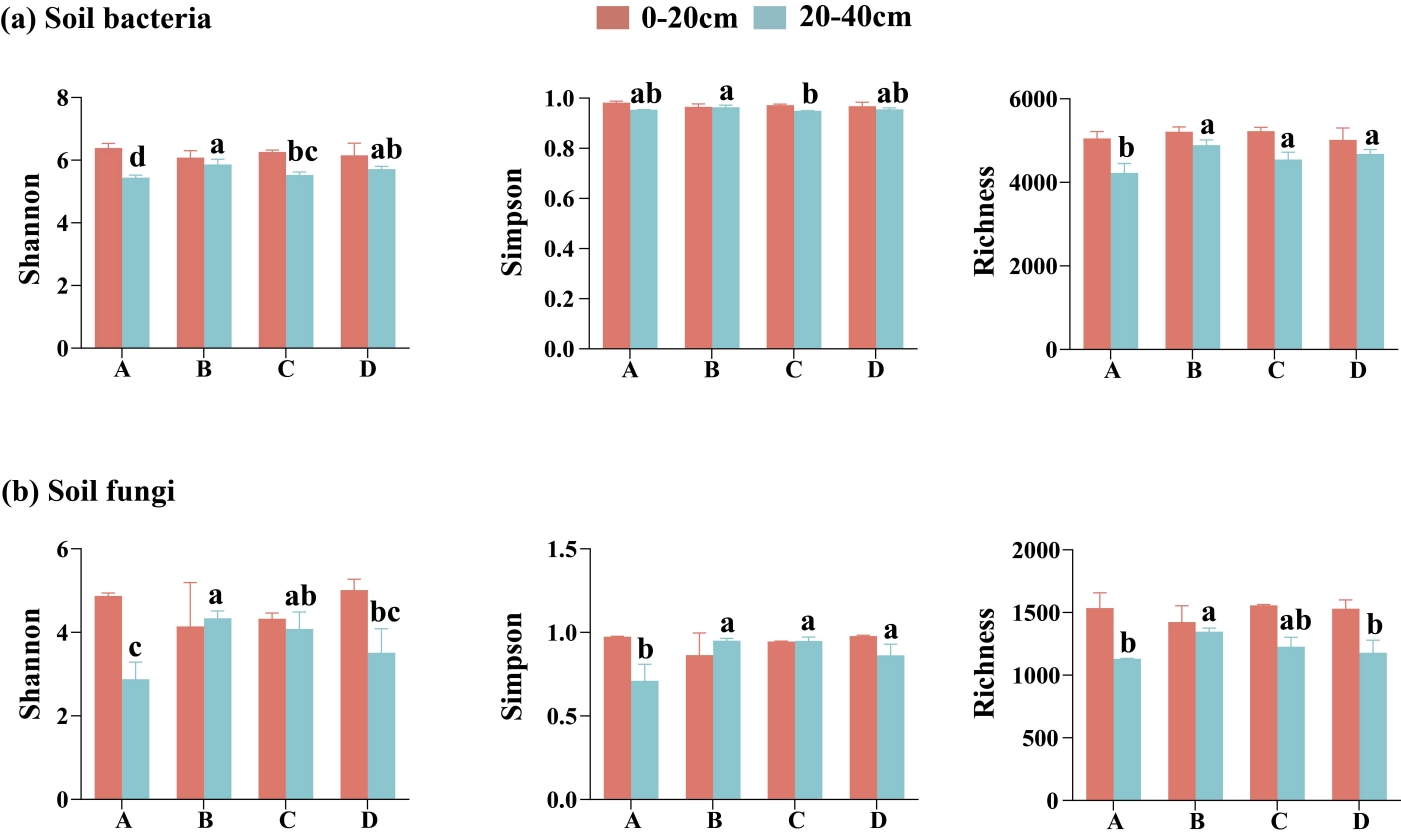
Changes in Shannon, Simpson, and Richness indices of soil bacterial (**a**) and fungal (**b**) communities in 0–20 and 20–40 cm soil layers at different restoration stages. Lowercase letters indicate significant differences among different restoration stages within the same soil depth, as determined by the LSD post hoc test (p<0.05).

Significant variation in soil bacterial and fungal α-diversity (Shannon, Simpson, and Richness) was observed across soil depth (p<0.05, Table S4). Across all restoration stages, α-diversity in the topsoil consistently exceeded that of the subsoil (Figure 4). In the topsoil, α-diversity remained relatively stable across restoration stages, whereas significant variations were observed in the subsoil. Notably, only bacterial richness was significantly influenced by the restoration stage (p<0.001). Overall, α-diversity followed a unimodal pattern, increasing initially and peaking during the broadleaf species invasion stage before declining thereafter. Correlation analysis revealed that bacterial α-diversity (Shannon, Simpson, and Richness) was significantly positively associated with multiple soil properties (TP, NP, TN, SOC, soil C:N). Fungal α-diversity exhibited a stronger positive association with AP (Figure S1).

**Figure 5:**
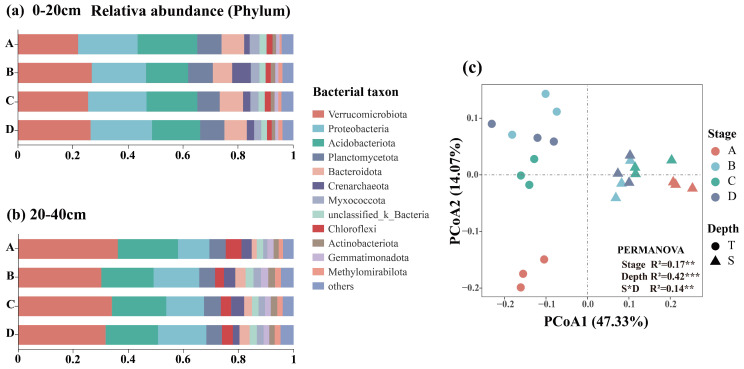
Phylum-level relative abundance of bacterial communities in soil layers of 0–20 (**a**) and 20–40 cm (**b**) at different restoration stages (showing only phyla > 1% in abundance). PCoA of bacterial communities based on Bray–Curtis distance (**c**).The effects of restoration stage, soil depth, and their interaction on bacterial community structure were assessed using PERMANOVA (*** *p* < 0.001, ** *p* < 0.01, * *p* < 0.05).

**Figure 6:**
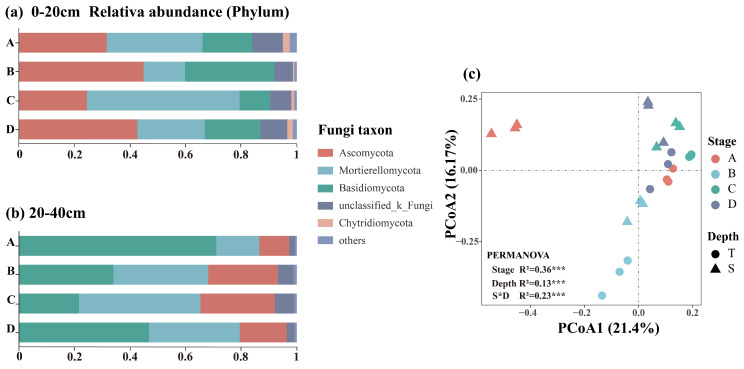
Phylum-level relative abundance of fungal communities in soil layers of 0–20 (**a**) and 20–40 cm (**b**) at different restoration stages (showing only phyla > 1% in abundance). PCoA of fungal communities based on Bray–Curtis distance (**c**). The effects of restoration stage, soil depth, and their interaction on fungal community structure were assessed using PERMANOVA (*** *p* < 0.001, ** *p* < 0.01, * *p* < 0.05).

PCoA revealed that the first two principal coordinates (PCoA1 and PCoA2) accounted for 61.40% and 37.6% of the total variation in bacterial and fungal communities, respectively (Figure 5c and Figure 6c). PERMANOVA further demonstrated that soil depth had a stronger influence on bacterial community structure ($R^{2}=0.42$, p<0.001), whereas both restoration stage and soil depth significantly affected fungal community structure (p<0.001), with the restoration process exerting a more pronounced impact on fungal communities ($R^{2}=0.36$, p<0.001).

### 3.3. Composition of Soil Bacteria and Fungi

Twelve bacterial phyla had relative abundances exceeding 1%. The three most dominant phyla were Verrucomicrobia (29%), Proteobacteria (18%), and Acidobacteria (19%) (Figure 5a,b), all of which were significantly influenced by soil depth (p<0.05, Table S5). In comparison with the topsoil, the relative abundances of Verrucomicrobia and Acidobacteria in the subsoil increased by approximately 7% and 2%, respectively, while the abundance of Proteobacteria decreased by 6%, thereby reducing its dominant status (Table S5). The relative abundance of Acidobacteriota was significantly affected by the restoration stage (p<0.001, Table S5). It exhibited higher abundance during the larch plantation stage, followed by a decline in relative abundance as restoration progressed.

Overall, the soil fungal community was primarily composed of members from Mortierellomycota (40%), Basidiomycota (32%), and Ascomycota (28%). The relative abundances of Ascomycota and Basidiomycota were significantly influenced by soil depth (p<0.05, Table S6). Ascomycota dominated the topsoil, whereas Basidiomycota became more abundant in the subsoil, thereby replacing Ascomycota as the dominant group. Restoration stage significantly influenced the relative abundance of Mortierellomycota (p<0.001, Table S6), with the highest abundance observed during the conifer–broadleaf competition stage. Additionally, Basidiomycota was most abundant during the larch plantation stage, followed by a decline and subsequent increase as restoration progressed. Ascomycota showed the greatest relative abundance during the broadleaf species invasion stage.

**Figure 7:**
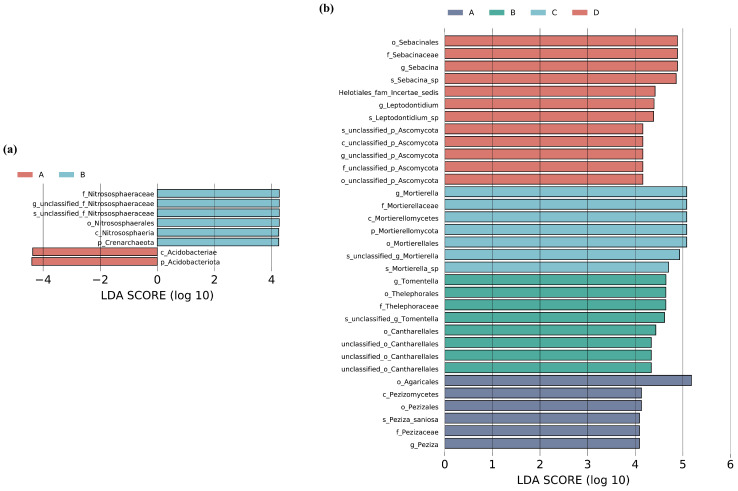
LEfSe analysis of soil bacteria (**a**) and fungi (**b**) across different restoration stages (LDA >4.0, p<0.05).

LEfSe analysis of bacterial communities across different restoration stages revealed that p_Acidobacteriota and c_Acidobacteriae were significantly enriched during the larch plantation stage (Figure 7a). In contrast, f_Nitrososphaeraceae, o_Nitrososphaerals, c_Nitrososphaeria, and p_Crenarchaeota showed significant enrichment during the broadleaf species invasion stage. For fungal communities (Figure 7b), LEfSe analysis showed that the broadleaf dominance stage was dominated by o_Sebacinales, f_Sebacinaceae, *g_Sebacina*, and *s_Sebacina*_sp. The conifer–broadleaf competition stage was characterized by a predominance of *g_Mortierella*, f_Mortierellaceae, and c_Mortierellomycota. During the broadleaf species invasion stage, *g_Tomentella*, o_Thelephorales, and f_Thelephoraceae were significantly enriched, whereas o_Agaricales was more abundant during the larch plantation stage.

### 3.4. Relationship Between Soil Properties, Litter Properties, and Soil Microbial Communities

**Figure 8:**
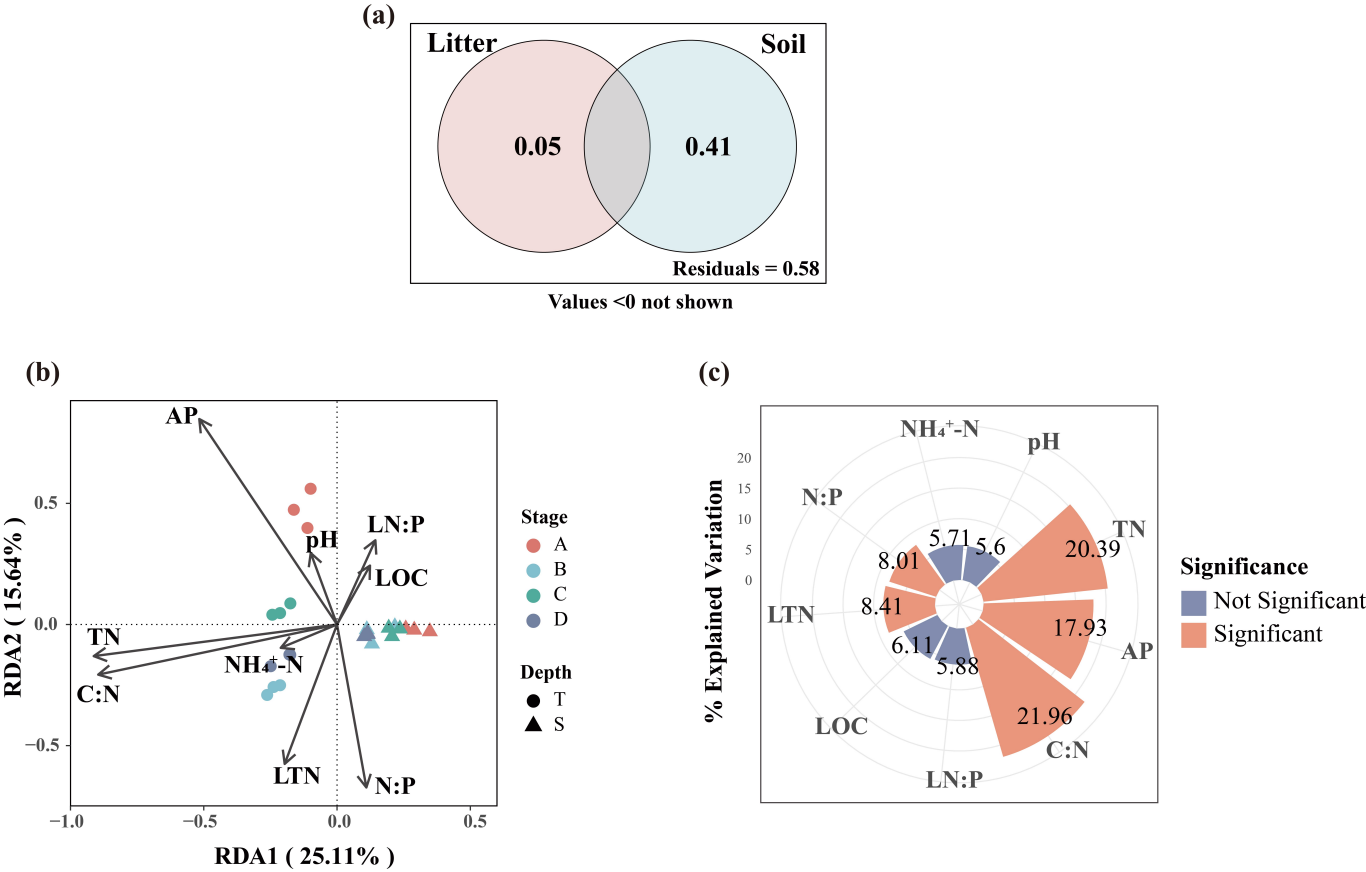
VPA shows the percentages of variation in bacterial communities explained by soil properties and litter properties (**a**). RDA of soil bacterial communities with soil and litter properties (**b**). Independent contributions and significance of each environmental factor analyzed using HP (**c**).

The VPA results indicate that litter properties and soil properties collectively explained 42% of the bacterial community variation. The independent contribution of soil properties (41%) was significantly greater than that of litter properties (5%), with negligible joint explanatory power between the two sets (Figure 8a). The RDA analysis indicated that the first and second constrained axes explained 25.11% and 15.64% of the variation in bacterial communities, respectively (Figure 8b). Together, the nine variables accounted for 47.9% of the total variance in bacterial communities. Hierarchical partitioning revealed the independent contributions of each factor, identifying the soil C:N (21.96%), TN (20.39%), and AP (17.93%) as the key drivers of bacterial community variation (Figure 8c).

**Figure 9:**
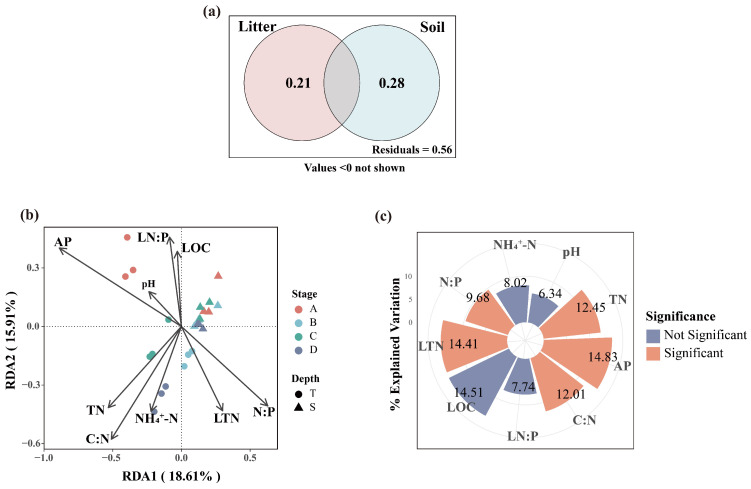
VPA shows the percentages of variation in fungal communities explained by soil properties and litter properties (**a**). RDA of soil fungal communities with soil and litter properties (**b**). Independent contributions and significance of each environmental factor analyzed using HP (**c**).

The VPA results indicated that litter properties and soil properties together explained 44% of the variation in fungal community structure. The independent contributions of litter properties was 21%, while that of soil properties was 28%, with a very small proportion of variation explained by their shared effects (Figure 9a). The RDA analysis indicated that the first and second constrained axes explained 18.61% and 15.91% of the variation in fungal community, respectively (Figure 9b). Together, the nine variables accounted for 48.3% of the total variance in fungal communities. Hierarchical partitioning revealed the independent contributions of each factor, identifying the AP (14.83%), LTN (14.41%), and TN (12.45%) as the key drivers of fungal community variation (Figure 9c).

**Figure 10:**
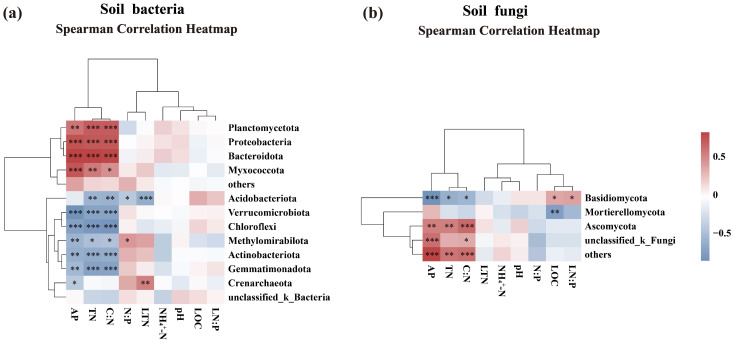
Spearman correlation analysis between soil properties, litter properties, and bacterial (**a**) and fungal (**b**) phyla with relative abundances > 1% (*** *p* < 0.001, ** *p* < 0.01, * *p* < 0.05).

Correlation heatmaps revealed distinct association patterns between soil bacterial and fungal communities and specific soil and litter properties (Figure 10). Verrucomicrobia exhibited a significant negative correlation with the AP, TN, and the soil C:N (p<0.001), whereas Proteobacteria showed strong positive correlations with these same variables (p<0.001). Acidobacteria exhibited significant negative associations with the soil C:N, the soil N:P, TN, and LTN (p<0.05, Figure 10a). Among soil fungi, Basidiomycota correlated positively with LOC and the litter N:P, but correlated negatively with AP, TN, and the soil C:N. Ascomycota showed significant positive correlations with the soil C:N, TN, and AP. Mortierellomycota correlated negatively with LOC (Figure 10b).

## 4. Discussion

### 4.1. Response of Litter and Soil Properties to Restoration Stages and Soil Depths

The forest restoration process is accompanied by changes in the aboveground vegetation types, which alter litter composition and chemical properties [30], in turn exerting an influence on other soil physicochemical characteristics [31]. Litter properties (LOC, LTN, and litter C:N) exhibited highly significant differences across various restoration stages. Notably, the litter C:N ratio decreased significantly from the high values observed in the larch plantation stage to the broadleaf species invasion stage, followed by a slight increase in the broadleaf dominance stage. This pattern reflects a shift in the quality of litter inputs and their decomposition rate during the restoration process [32]. Such dynamics are closely associated with the increased proportion of broadleaf species, such as *J. mandshurica* and *U. davidiana*, and their distinct litter characteristics during succession. Broadleaf tree litter typically exhibits a low C:N ratio, which often enhances its decomposability [22,33]. In contrast, coniferous tree litter usually contains higher levels of carbon-rich lignin and tannins, and its litter quality is lower (with a higher C:N ratio), resulting in a decomposition rate that is generally slower than that of broadleaf tree litter [34,35,36]. In the broadleaf dominance stage, the litter C:N ratio increases, possibly due to a higher proportion of coarse litter residues with high lignin content.

Our study demonstrates that soil nutrient levels decline with increasing depth, a pattern consistent with findings from previous research [37,38,39]. This phenomenon is attributed to the enrichment of litter input and biological debris in the topsoil [37], which increases its nutrient content. We observed a gradual decline in the content of TP, NO_3_^−^-N, and AP in the topsoil during the restoration of larch plantations, while these nutrient indicators showed a progressive increase in the subsoil. This pattern is primarily driven by interactions within the vegetation–soil system and the resulting nutrient redistribution processes. On one hand, as restoration progresses, the increase in vegetation biomass and the complexity of plant communities can lead to enhanced nutrient uptake by vegetation, significantly depleting TP, NO_3_^−^-N, and AP in the topsoil. A considerable portion of these absorbed nutrients may be fixed in the growing plant tissues. On the other hand, vegetation recovery improves soil structure [40], which enhances water infiltration and leaching, facilitating the downward migration of soluble nutrients and their gradual accumulation in the subsoil [41]. During the restoration of larch plantations, soil nutrients also exhibit significant stage-specific variations. These dynamics are likely influenced, both directly and indirectly, by community structure and litter decomposition within the stand [42,43]. Specifically, soils in the larch plantation stage exhibited lower levels of TN and SOC, primarily due to the input of low-quality coniferous litter, which slows nutrient turnover [44]. During the broadleaf species invasion stage, SOC, TN, and stoichiometric ratios all reached peak levels, primarily due to significantly improved litter quality resulting from mixed litter inputs, which accelerated organic matter mineralization and nutrient release. The decrease in soil nutrient content observed during the conifer–broadleaf competition stage and the broadleaf dominance stage is likely due to the increased nutrient demand of more complex vegetation communities, leading to the continuous transfer and accumulation of nutrients into the vegetation pool [45].

### 4.2. Changes in Microbial Diversity and Influencing Factors Across Restoration Stages and Soil Depths

This study observed significantly higher microbial α-diversity in topsoil compared to subsoil, which is consistent with most previous studies [46,47,48]. This pattern may be attributed to the better aeration and greater microbial activity in surface soil, which are conducive to enhanced microbial diversity [48]. Significant variation in bacterial and fungal α-diversity across restoration stages was detected exclusively in the subsoil, whereas topsoil diversity remained relatively stable throughout the restoration process. This suggests that microbial community diversity in subsoil is more responsive to environmental changes induced by restoration processes. One likely explanation is that surface soils maintain relatively stable conditions due to consistently higher nutrient availability, which provides a steady energy supply and supports a more resilient microbial community. In contrast, subsoils are typically colder, more humid, and oxygen-limited, resulting in slower organic matter decomposition and lower nutrient availability. These conditions are more likely to limit microbial growth [49,50], thereby amplifying the impact of environmental changes on microbial α-diversity. Bacterial richness responds sensitively to restoration stages, likely because changes in aboveground vegetation types modify the soil environment [20,51,52], thereby exerting new selective pressures. These pressures particularly affect bacteria that are nutrient-sensitive and have short reproductive cycles, ultimately leading to significant changes in bacterial richness.

In our study, bacterial α-diversity (Shannon, Simpson, and Richness) exhibited strong positive relationships with soil TN, SOC, and C:N (Figure S1a). Overall, bacterial α-diversity was highest during the broadleaf species invasion stage and exhibited a declining trend during the restoration process, mirroring the changes in these soil properties. This finding highlights the importance of soil characteristics in explaining variations in bacterial diversity. Notably, the positive correlation between bacterial α-diversity and the soil C:N observed in our study contrasts with earlier research suggesting that bacterial communities tend to favor high-quality soils for growth and reproduction [53]. One possible explanation is that in environments with a high C:N ratio, microbes must adopt more refined strategies to compete for limited nitrogen resources. This may lead to increased niche differentiation, allowing more species to coexist by utilizing different resources, thereby increasing overall diversity. Previous studies have identified soil pH as the primary factor influencing fungal α-diversity [54]. However, we did not detect a significant association between soil pH and fungal α-diversity (Figure S1b). This discrepancy may be attributed to the relatively higher soil pH (5.3–6.1) at our study site, as current evidence for a significant link between fungal α-diversity and soil pH primarily comes from strongly acidic (4.5–5.5) soil [19,55]. A more pronounced positive correlation was found between fungal α-diversity and AP, suggesting that AP is likely a primary factor influencing fungal diversity at our site. AP likely influences fungal diversity by altering phosphorus availability in the soil [56].

### 4.3. Changes in Microbial Community Composition Across Restoration Stages and Soil Depths

The dominant bacterial phyla across all restoration stages were consistently Verrucomicrobia, Proteobacteria, and Acidobacteria. Among these, Proteobacteria and Acidobacteria represent major bacterial groups commonly found in soils supporting forested vegetation [57,58,59]. Proteobacteria typically thrive in oxygen-rich environments with high organic matter content [48], which well accounts for their marked reduction in relative abundance and diminished dominance in the subsoil. Furthermore, consistent with previous findings [60,61], we observed an increasing trend in the relative abundance of Proteobacteria alongside a decline in Acidobacteria during the restoration process. Many members within the Proteobacteria phylum exhibit copiotrophic strategies [62], whose abundance significantly increases when readily decomposable substrates become more available in the soil. Thus, the increase in the relative abundance of Proteobacteria is likely attributable to the accumulation of carbon and nitrogen from litter decomposition and root exudates, resulting from increased above- and belowground biomass as restoration progresses, which aligns with their r-strategy. In contrast, Acidobacteria as a phylum are generally associated with oligotrophic conditions and are better adapted to nutrient-poor environments [62,63]. This is consistent with the LEfSe analysis results, which showed that the larch plantation stage, characterized by low soil nutrient content, exhibited significant enrichment of Acidobacteria and its class Acidobacteriia. Additionally, during the broadleaf invasion stage, Nitrososphaeraceae, a family associated with nitrification, was significantly enriched [64], indicating an enhancement of soil nitrogen cycling at this stage.

Basidiomycota exhibited markedly greater abundance in the subsoil than in the topsoil. This distribution pattern likely reflects its ecological adaptation to cooler and less moist conditions [65]. Moreover, most genera within Basidiomycota typically form symbiotic associations with plant roots [66], allowing them to directly acquire carbon from host plants. The higher root density in deeper soil layers likely contributes to their increased relative abundance in the subsoil, allowing Basidiomycota to replace Ascomycota as the dominant fungal group. Basidiomycota encompasses a variety of ectomycorrhizal and saprophytic species that significantly contribute to the degradation of resistant organic compounds [17,67]. We found that Basidiomycota were most abundant in the larch plantation stage, then decreased initially and subsequently increased as the restoration progressed. This trend aligns with the pattern of litter quality, reflecting the adaptive response of Basidiomycota fungi to changes in litter substrate quality. Distinct fungal taxa were characteristic across different restoration stages. In the larch plantation stage, order Agaricales was notably enriched; many fungi within this order possess saprotrophic capabilities, which are likely linked to the need for organic matter decomposition associated with needle litter accumulation. During the broadleaf invasion stage, family Thelephoraceae and genus *Tomentella* were significantly enriched, likely due to the invading broadleaf trees relying on ectomycorrhizal associations to expand their nutrient acquisition networks [68]. During the broadleaf dominance stage, the enrichment of genus *Sebacina* may facilitate mycorrhizal network formation with key species, thereby supporting community stability.

### 4.4. Relationships Between Microbial Community and Soil and Litter Properties

This study demonstrates that both soil and litter properties are important factors driving the spatiotemporal variation of soil microbial communities during the restoration of larch plantations. Consistent with our second hypothesis, soil properties play a dominant role in structuring both bacterial and fungal communities, largely because soil provides the primary habitat as well as a major source of carbon and nutrients for microorganisms. In contrast, litter influences microbial communities mainly by altering soil physical conditions and chemical composition [69].

Our study further found that soil TN and AP were important factors shaping bacterial and fungal communities, which is consistent with previous studies [8,70,71,72]. Previous research has demonstrated that the availability of soil nutrients shapes microbial community structure and composition [73,74]. Nitrogen is an essential nutrient for microbial growth and development [75]. In the present study, dominant bacteria including Proteobacteria, Acidobacteria, and Verrucomicrobia, as well as the predominant fungal phyla Ascomycota and Basidiomycota, all showed significant correlations with TN content, indicating their sensitivity to soil nitrogen levels. AP influences microbial composition by altering phosphorus availability in the soil. Proteobacteria show a significant correlation with AP content, employing efficient phosphorus uptake and utilization strategies that enable rapid proliferation under high-AP conditions, thereby dominating the community. In contrast, Acidobacteria may be better adapted to low-phosphorus environments, and their abundance tends to decrease as AP levels rise. Within the fungal community, many saprotrophic members of Ascomycota produce abundant extracellular enzymes, such as acid phosphatases, to mineralize organic phosphorus. Their fast growth and high phosphorus acquisition efficiency allow them to thrive in environments rich in organic phosphorus. On the other hand, Basidiomycota, which includes a large number of ectomycorrhizal fungi, employs ecological strategies more suited to absorbing and mobilizing phosphorus in nutrient-poor soils, making them more competitive under low-phosphorus conditions.

Consistent with previous research [76], we found that litter properties contributed more strongly to variations in soil fungal communities than to bacterial communities, which also supports our third hypothesis. This may be attributed to the fact that in the early stages of larch plantation restoration, the litter primarily consists of recalcitrant coniferous needles. Fungi, which can secrete a variety of extracellular enzymes, play a pivotal role in decomposing such refractory plant litter [77]. Their community composition is likely adapted to the chemical properties of this specific substrate. As restoration proceeds and coniferous litter transitions toward mixed litter, fungi are expected to respond more sensitively to changes in litter quality and undergo a succession toward taxa that are more efficient at utilizing mixed litter substrates. Hierarchical partitioning further revealed that LTN (14.41%) was an important factor influencing soil fungal community structure. Litter serves as a critical nitrogen source for fungi, which is essential for their survival and activity, as nitrogen is required to synthesis of vital macromolecules such as proteins and nucleotides [59]. Furthermore, the inherently high C:N ratio of litter in larch plantations results in low nitrogen availability during decomposition, creating a limiting factor for fungal community activity. In the initial low-nitrogen environment, Basidiomycota, which are capable of decomposing complex organic compounds, became enriched. Subsequently, the invasion of broadleaf species increased nitrogen levels and availability in the litter layer. This shift induced changes in the fungal community structure, favoring the enrichment of Ascomycota, an r-strategist taxa.

## 5. Conclusions

This study comprehensively investigated how restoration stage and soil depth influence the diversity and composition of soil bacterial and fungal communities in larch plantations, as well as clarifying the relative contributions of soil and litter properties in shaping microbial community dynamics. Results revealed that the α-diversity (Shannon, Simpson, and Richness) of both bacteria and fungi remained relatively stable in the topsoil but varied significantly across successional stages in the subsoil, with the broadleaf species invasion stage exhibiting the highest α-diversity. Compared to the larch plantation stage, the relative abundance of Proteobacteria and Ascomycota increased in the broadleaf dominance stage, while that of Acidobacteria and Basidiomycota decreased. The restoration process had a stronger influence on fungal communities, whereas bacterial communities showed greater spatial dependency compared to fungal communities. More importantly, we identified soil properties, especially AP and TN, as the dominant factors driving the structural variations in both bacterial and fungal communities, whereas litter properties played a more substantial regulatory role in shaping fungal community composition.

Our study clearly demonstrates a restoration trajectory of the larch plantation toward a community dominated by native broadleaf species. Based on our findings, we emphasize the importance of jointly considering both soil and litter properties to gain a more comprehensive understanding of microbial community ecology under changing environmental gradients. We further recommend that in the ecological restoration and management of larch plantations in Northeastern China, greater attention should be paid to regulating litter quality and monitoring the dynamics of soil AP, TN, and fungal communities to facilitate efficient ecosystem restoration. Additionally, long-term monitoring of the natural succession trajectory of larch plantations is still needed in the future.

## Data Availability

The original contributions presented in this study are included in the article/Supplementary Materials. Further inquiries can be directed to the corresponding author.

## Supplementary Materials

The following supporting information can be downloaded at https://www.mdpi.com/article/10.3390/microorganisms13102359/s1. Figure S1: Spearman correlation analysis between α-diversity indices of bacteria (a) and fungi (b) and soil and litter properties; Table S1: Description of the sampling plots; Table S2: Changes in litter properties at different restoration stages; Table S3: Changes in soil properties of the 0–20 and 20–40 cm soil layers at different restoration stages; Table S4: Changes in soil microbial α-diversity at different restoration stages; Table S5: Phylum-level relative abundance of bacterial communities in soil layers of 0–20 and 20–40 cm at different restoration stages; Table S6: Phylum-level relative abundance of fungal communities in soil layers of 0–20 and 20–40 cm at different restoration stages.
