# Supplementary material for: Relative Contributions of Soil and Litter Properties to Soil Microbial Community Variations During the Restoration of Larch Plantations to Mixed Forests
Source: Microorganisms. 2025 Oct 14;13(10):2359. doi: 10.3390/microorganisms13102359 (PMC12565900; doi:10.3390/microorganisms13102359)
Supplement: Supplementary file 1 [file microorganisms-13-02359-s001.zip › Supplementary Materials.pdf]

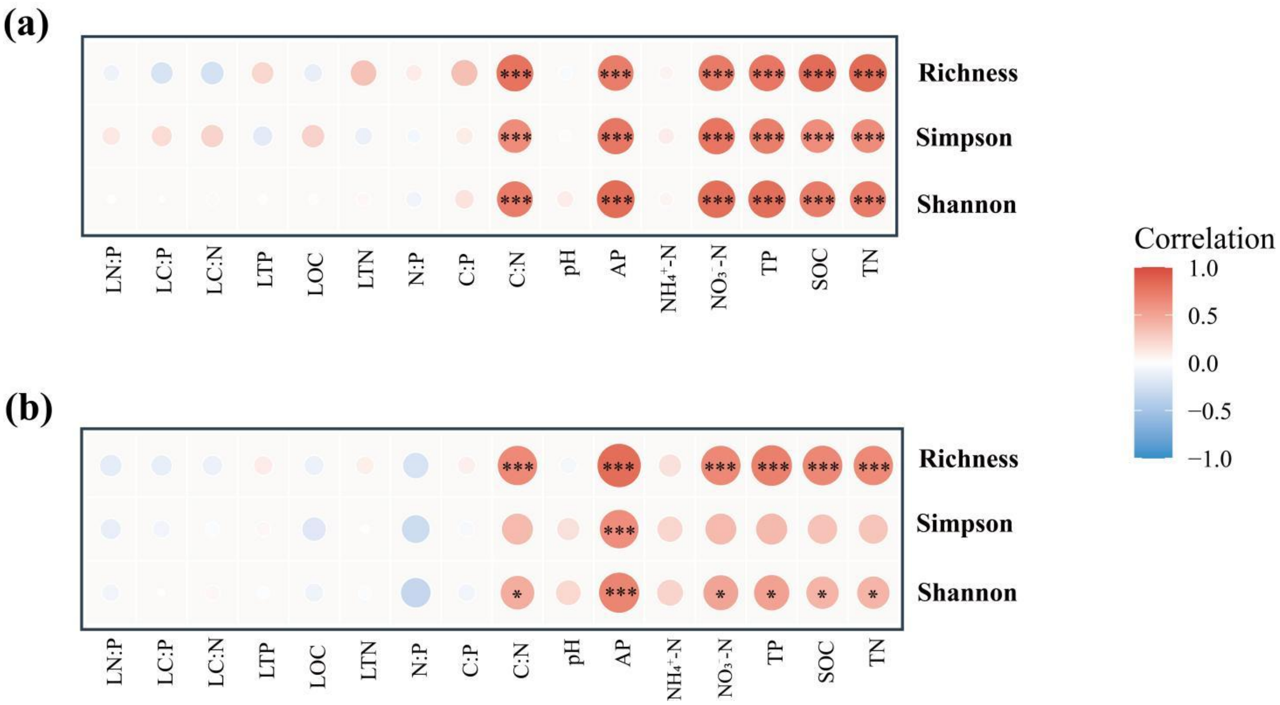

Figure S1. Spearman correlation analysis between α-diversity indices of bacteria (**a**) and fungi (**b**) and soil and litter properties.

Table S1. Description of the sampling plots.

Site	Altitude(m)	Slope(°)	Latitude and longitude	Main tree species	Features
A	301	5	46.73805355°N 129.09348389°E	*Larix gmelinii*	The stand exhibits a typical mono-dominant community structure. The arbor layer consists of a pure larch plantation, characterized by uniform tree distribution and a single age class.
B	368	6	46.72166223°N 129.05870377°E	*Larix gmelinii, Ulmus davidiana, Juglans mandshurica*	A subcanopy layer has formed, with larch remaining the dominant species in the main canopy, while broadleaf species have successfully established and formed a secondary tree layer.
C	287	5	46.67239408°N 129.08094867°E	*Juglans mandshurica, Ulmus davidiana, Larix gmelinii, Syringa reticulata*	A stratified mixed stand. Larch and broad-leaved trees jointly form the main canopy layer, with their importance values being comparable. Competition is intense.
D	351	4	46.74037421°N 129.10100221°E	*Juglans mandshurica, Syringa reticulata, Ulmus davidiana,*	Broadleaf species dominate absolutely, forming a broadleaf canopy. The importance value of larch has significantly decreased.

A, larch plantation stage; B, broadleaf species invasion stage; C, conifer–broadleaf competition stage; D, broadleaf dominance stage

Table S2. Changes in litter properties at different restoration stages.

Stage	A	B	C	D	One-way ANOVA
LOC g/kg	374.43±2.284a	360.70±1.553b	320.60±2.524c	345.40±0.850d	**<0.001**
LTN g/kg	14.97±0.267d	23.43±0.219a	21.87±0.186b	18.70±0.115c	**<0.001**
LTP g/kg	0.81±0.163b	1.59±0.172a	1.83±0.085a	1.34±0.183a	0.091
LC:N	25.02±0.498a	15.40±0.109c	14.68±0.086c	18.50±0.141b	**<0.001**
LN:P	19.59±2.992a	15.08±1.792ab	11.99±0.579b	14.44±1.752ab	0.126
LC:P	493.51±83.577a	231.86±26.033b	175.77±7.438b	266.83±32.440b	**0.006**

Data are presented as mean ± standard error (SE), n = 3. Different lowercase letters indicate significant differences in litter properties among restoration stages ($p < 0.05$). A, larch plantation stage; B, broadleaf species invasion stage; C, conifer–broadleaf competition stage; D, broadleaf dominance stage. LOC, litter organic carbon; LTN, litter total nitrogen; LTP, litter total phosphorus; LC:N, litter carbon to nitrogen ratio; LN:P, litter nitrogen to phosphorus ratio; LC:P, litter carbon to phosphorus ratio.

Table S3. Changes in soil properties of the 0-20 and 20-40 cm soil layers at different restoration stages.

Stage	A		B		C		D		Two-wayANOVA		
	0-20cm	20-40cm	0-20cm	20-40cm	0-20cm	20-40cm	0-20cm	20-40cm	Stage	Depth	S*D
SOC g/kg	140.63±0.120d	40.3±2.354c	298.90±1.656a	71.27±0.491a	235.73±5.750b	32.80±0.100d	163.10±2.950c	65.47±0.546b	**<0.001**	**<0.001**	**<0.001**
TN g/kg	10.73±0.067c	3.70±0.058b	19.00±0.100a	5.57±0.067a	15.83±0.296b	3.37±0.033c	10.93±0.203c	5.37±0.133a	**<0.001**	**<0.001**	**<0.001**
TP g/kg	2.73±0.149d	0.40±0.025	1.58±0.106a	0.43±0.049	1.54±0.097b	0.42±0.032	1.23±0.056c	0.49±0.036	**<0.001**	**<0.001**	**<0.001**
NO_3^--N ug/g	315.91±5.451a	25.19±9.923bc	286.06±9.893ab	44.60±2.477b	261.62±14.404b	15.25±0.662c	141.64±0.611c	70.71±6.833a	**<0.001**	**<0.001**	**<0.001**
NH_4^+-N ug/g	13.56±3.653	23.17±3.499a	13.87±4.740	10.22±1.720b	21.27±3.591	10.63±1.492b	24.76±2.731	12.37±1.854b	0.168	0.069	**0.011**
pH	6.14±0.445a	5.65±0.012	5.73±0.152ab	5.70±0.057	5.28±0.012b	5.67±0.010	5.93±0.037ab	5.73±0.055	0.103	0.509	0.108
AP mg/kg	81.33±4.853a	7.07±1.717c	18.36±0.496c	20.37±1.672a	44.40±0.378b	10.82±0.718bc	23.35±0.780c	14.22±0.378b	**<0.001**	**<0.001**	**<0.001**
C:N ratio	13.10±0.104c	10.97±0.491b	15.72±0.088a	12.80±0.106a	14.86±0.085b	9.72±0.110c	14.90±0.026b	12.20±0.156a	**<0.001**	**<0.001**	**<0.001**
N:P ratio	3.96±0.242c	9.23±0.704b	12.13±0.865a	13.40±1.783a	10.35±0.649ab	8.11±0.699b	8.92±0.307b	11.15±0.824ab	**<0.001**	**0.018**	**0.005**
C:P ratio	51.84±2.955c	100.94±11.702bc	190.87±13.760a	171.13±21.276a	154.05±9.243b	79.02±6.718c	133.13±4.770b	136.07±9.443b	**<0.001**	0.202	**<0.001**

Data are presented as mean ± standard error (SE), n = 3. Different lowercase letters indicate significant differences in soil physicochemical properties among different restoration stages within the same soil layer ($p < 0.05$). The P values in bold indicate statistically significant differences among the restoration stage, soil depth or their interaction using two-way ANOVA ($p < 0.05$). A, larch plantation stage; B, broadleaf species invasion stage; C, conifer–broadleaf competition stage; D, broadleaf dominance stage. SOC, soil organic carbon; TN, total nitrogen; TP, total phosphorus; NO_3^--N, nitrate nitrogen; NH_4^+-N, ammonium nitrogen; AP, available phosphorus; C:N, soil carbon to nitrogen ratio; N:P, soil nitrogen to phosphorus ratio; C:P, soil carbon to phosphorus ratio.

Table S4. Changes in soil microbial α-diversity at different restoration stages.

Stage	Soil bacteria			Soil Fungi		
	**Shannon**	**Simpson**	**Richness**	**Shannon**	**Simpson**	**Richness**
**0-20cm**						
A	6.39±0.086	0.98±0.003	5050.00±98.083	4.87±0.039	0.97±0.002	1535.00±70.406
B	6.08±0.128	0.97±0.007	5213.67±66.968	4.14±0.608	0.87±0.076	1424.00±74.702
C	6.26±0.037	0.97±0.002	5226.67±53.695	4.33±0.078	0.95±0.001	1557.00±4.000
D	6.16±0.222	0.97±0.009	5018.33±163.907	5.02±0.148	0.98±0.003	1531.67±40.704
**20-40cm**						
A	5.44±0.046d	0.95±0.001ab	4226.67±129.333b	2.88±0.239c	0.71±0.057b	1130.67±1.764b
B	5.86±0.095a	0.96±0.005a	4892.67±72.075a	4.34±0.100a	0.95±0.007a	1347.33±16.597a
C	5.53±0.053bc	0.95±0.000b	4546.00±100.550a	4.08±0.234ab	0.95±0.013a	1227.00±43.313ab
D	5.71±0.049ab	0.96±0.003ab	4683.67±61.282a	3.51±0.334bc	0.86±0.038a	1179.33±57.869b
**Two-way ANOVA**						
Stage	0.899	0.584	**＜0.001**	0.493	0.825	0.572
Depth	**＜0.001**	**＜0.001**	**0.007**	**＜0.001**	**0.015**	**＜0.001**
S*D	**0.018**	0.219	0.052	**0.003**	**0.041**	**0.0142**

Data are presented as mean ± standard error (SE), n = 3. Different lowercase letters indicate significant differences in microbial alpha-diversity (Shannon, Simpson, and Richness indices) among restoration stages within the same soil layer ($p < 0.05$). The P values in bold indicate statistically significant differences among the restoration stage, soil depth or their interaction using two-way ANOVA ($p < 0.05$). A, larch plantation stage; B, broadleaf species invasion stage; C, conifer–broadleaf competition stage; D, broadleaf dominance stage.

Table S5. Phylum-level relative abundance of bacterial communities in soil layers of 0-20 and 20-40 cm at different restoration stages.

Stage	*Verrucomicrobiota*	*Acidobacteriota*	*Proteobacteria*	*Planctomycetota*	*Bacteroidota*	*Crenarchaeota*	*Chloroflexi*	*Myxococcota*	*unclassified_k_Bacteria*	*Actinobacteriota*	*Gemmatimonadota*	*Methylomirabilota*	*others*
**0-20 cm**													
A	0.22 ± 0.019	0.22 ±0.012a	0.22 ±0.014	0.09 ± 0.007	0.08±0.011	0.02±0.004b	0.02 ± 0.001	0.03 ± 0.003a	0.03 ± 0.002	0.01 ±0.001	0.01 ±0.001	0.01 ±0.000b	0.04 ±0.003
B	0.27 ± 0.031	0.15 ±0.004b	0.20 ±0.018	0.09 ± 0.014	0.07±0.008	0.07±0.013a	0.02 ± 0.001	0.03 ± 0.005ab	0.02 ± 0.002	0.02 ±0.002	0.01 ±0.002	0.01 ±0.003a	0.04 ±0.005
C	0.25 ± 0.010	0.18 ±0.004ab	0.21 ±0.012	0.08 ± 0.004	0.08±0.006	0.03±0.004b	0.02 ± 0.002	0.03 ± 0.002ab	0.02 ± 0.001	0.01 ±0.001	0.01 ±0.001	0.01 ±0.000ab	0.04 ±0.001
D	0.26 ± 0.030	0.17 ±0.015b	0.22 ±0.023	0.09 ± 0.005	0.08±0.011	0.03±0.002b	0.02 ± 0.001	0.03 ± 0.001b	0.02 ± 0.001	0.01 ±0.002	0.01 ±0.001	0.02 ±0.000a	0.04 ±0.003
**20-40 cm**													
A	0.36±0.007a	0.22±0.009a	0.11±0.008c	0.06±0.002	0.02±0.002c	0.04±0.002ab	0.06±0.003a	0.02±0.002b	0.02±0.001b	0.02±0.001	0.02±0.001	0.02±0.001b	0.04±0.002b
B	0.30±0.016c	0.19±0.003b	0.17±0.014ab	0.06±0.002	0.04±0.002a	0.04±0.006a	0.03±0.002b	0.03±0.000a	0.03±0.001a	0.03±0.002	0.03±0.003	0.02±0.001a	0.05±0.002ab
C	0.34± 0.011ab	0.20±0.004ab	0.14±0.004bc	0.06±0.002	0.03±0.004b	0.05±0.013a	0.04±0.008b	0.02±0.003a	0.02±0.000b	0.02±0.002	0.02±0.002	0.02± 0.001ab	0.04±0.001ab
D	0.32± 0.011bc	0.19±0.009b	0.18±0.014a	0.06±0.004	0.04±0.001ab	0.02±0.004b	0.04±0.002b	0.02±0.001a	0.03± 0.001ab	0.02±0.003	0.02±0.003	0.02± 0.003ab	0.05±0.005a
**Two-way ANOVA**													
Stage	0.922	**< 0.001**	0.146	0.993	0.592	**0.001**	0.582	0.341	0.374	0.083	0.930	**0.002**	0.774
Depth	**< 0.001**	**0.012**	**< 0.001**	**< 0.001**	**< 0.001**	0.718	**< 0.001**	**< 0.001**	**0.029**	**< 0.001**	**< 0.001**	**< 0.001**	0.439
S*D	**0.049**	0.28	0.107	0.893	0.146	**0.005**	0.835	**0.010**	**0.010**	0.699	0.864	0.852	0.106

Data are presented as mean ± standard error (SE), n = 3. Different lowercase letters indicate significant differences in bacterial relative abundance among restoration

stages within the same soil layer (p < 0.05). The P values in bold indicate statistically significant differences among the restoration stage, soil depth or their interaction using two-way ANOVA (p < 0.05). A, larch plantation stage; B, broadleaf species invasion stage; C, conifer–broadleaf competition stage; D, broadleaf dominance stage.

Table S6. Phylum-level relative abundance of fungal communities in soil layers of 0-20 and 20-40 cm at different restoration stages.

Stage	*Basidiomycota*	*Mortierellomycota*	*Ascomycota*	*unclassified_k_Fungi*	*others*
**0-20cm**					
A	0.18±0.010b	0.35±0.017b	0.32±0.008	0.11±0.018a	0.05±0.014a
B	0.33±0.086a	0.15±0.035d	0.44±0.131	0.07±0.016b	0.01±0.003b
C	0.11±0.003b	0.55±0.010a	0.24±0.013	0.08±0.004ab	0.02±0.004b
D	0.20±0.013ab	0.24±0.021c	0.43±0.031	0.10±0.012ab	0.03±0.003ab
**20-40cm**					
A	0.71±0.039a	0.16±0.023b	0.11±0.014c	0.02±0.004b	0.01±0.001b
B	0.34±0.061bc	0.34±0.075ab	0.25±0.007a	0.06±0.016ab	0.01±0.001a
C	0.21±0.045c	0.44±0.070a	0.27±0.029a	0.07±0.020a	0.01±0.002ab
D	0.46±0.111b	0.34±0.090ab	0.17±0.014b	0.03±0.007ab	0.01±0.002ab
**Two-way ANOVA**					
Stage	0.089	**<0.001**	0.552	0.822	0.936
Depth	**0.003**	0.900	**<0.001**	**<0.001**	**<0.001**
S*D	0.400	**0.007**	0.094	**0.0134**	0.225

Data are presented as mean ± standard error (SE), n = 3. Different lowercase letters indicate significant differences in fungal relative abundance among restoration stages within the same soil layer (p < 0.05). The P values in bold indicate statistically significant differences among the restoration stage, soil depth or their interaction using two-way ANOVA (p < 0.05). A, larch plantation stage; B, broadleaf species invasion stage; C, conifer–broadleaf competition stage; D, broadleaf dominance stage.
